# Subwavelength Silicon Nanoblocks for Directional Emission Manipulation

**DOI:** 10.3390/nano10061242

**Published:** 2020-06-26

**Authors:** Tianyue Zhang, Xuewei Li, Jian Xu, Xiaoming Zhang, Zi-Lan Deng, Xiangping Li

**Affiliations:** 1 Guangdong Provincial Key Laboratory of Optical Fiber Sensing and Communications, Institute of Photonics Technology, Jinan University, Guangzhou 510632, China; kris1106@stu2019.jnu.edu.cn (X.L.); yusheng370@163.com (J.X.); zilandeng@jnu.edu.cn (Z.-L.D.); xiangpingli@jnu.edu.cn (X.L.); 2 College of Physics Science and Engineering Technology, Yichun University, Yichun 336000, China

**Keywords:** unidirectional emission, all-dielectric nanoantennas, fluorescence, nanophotonics

## Abstract

Manipulating the light emission direction and boosting its directivity have essential importance in integrated nanophotonic devices. Here, we theoretically propose a single dielectric silicon nanoblock as an efficient, multifunctional and ultracompact all-dielectric nanoantenna to direct light into a preferential direction. Unidirectional scattering of a plane wave as well as switchable directive emission fed by a localized emitter are demonstrated within the nanoantenna. The high directionalities are revealed to originate from a variety of mechanisms that can coexist within a single nanoblock, which contribute to the far-field radiation patterns of the outcoming light, thanks to the wealth of multipolar electric and magnetic resonances. The efficient beam redirections are also observed, which are sensitive to the local configurations of the emitter antenna coupled system. The designed antenna, with extreme geometry simplicity, ultracompact and low-loss features, could be favorable for highly sensitive sensing as well as applications in optical nanocircuits.

## 1. Introduction

Optically resonant nanostructures made from all-dielectric, high-refractive-index materials offer a versatile platform to realize nanoscale light manipulation [1]. Owing to the excitation of multipolar Mie-type resonances, dielectric nanostructures provide more degrees of freedom to design optical nanoantennas for controlling light emission [2,3,4,5]. One of the emerging and rapidly developing research branches is to control and boost the directivity of light emission, for the purpose of improving the collection efficiency [6]. One category of designed directive antennas is to display unidirectional scattering upon plane wave illumination, when the induced multipolar modes excited by plane waves are properly balanced (Kerker conditions) [7,8]. Another major category emphasizes the near-field excitation of the nanoantenna by a localized nearby emitter [9,10]. In such a context, the induced electric and magnetic modes that are coherent with respect to the source emitter will have specific phase relations, and thus can interfere in the far field, leading to the emission of the coupled emitter-antenna system with a controllable and predefined directivity. 

Studies have shown the unique capability to create unidirectional light radiation in multiple architectures, including single-element nanospheres [8,9], nanodisks [11,12,13,14,15], nanowires (cylinders) [16,17,18,19], rectangular patches [20], V-shaped [21] and stair-shaped nanoantennas [22], as well as multi-element array nanoantennas [23,24,25,26]. There are also many efforts in the field to achieve bi- or multi-directional scattering/emission [9,13,15,21,23,27,28]. In such a context, all-dielectric antennas are typically designed with broad Mie resonances, which allow for spectral overlaps between different multipolar modes at multi-wavelength positions. In addition, interferences in the optically induced multipolar resonances and the near-field emitter could have different amplitude and phase relationships, hence it is also possible to switch the directivity between the forward and the backward direction by simply varying the emitter-antenna distances or modifying the emission wavelength of the emitter. However, in contrast to those mentioned directive all-dielectric nanoantennas, detailed investigations of subwavelength nanoblocks have been seldom reported [29]. Although nanoblocks share similar geometric characteristics with nanocylinders, rectangular dielectric structures are of the most interest as building blocks for on-chip integrated devices due to the ease of fabrication through the semiconductor manufacturing process. Moreover, owing to the reduced symmetries, the electromagnetic resonances of nanoblocks can be tailored by varying the length, width and height, exhibiting richer properties than those of cylinders. Leveraging these attractive features to explore the directional light radiation mediated by subwavelength scale all-dielectric nanoblocks for a desired application is a worthwhile new endeavor. Moreover, to design ultracompact nanoantennas that integrate the functionality of emission direction control for both near and far field excitations is of great significance.

In this article, we carry out theoretical calculations to demonstrate the feasible manipulation of directive light emission from a single subwavelength silicon (Si) nanoblock. We not only study its directional scattering upon standard plane waves, but also the distinct switchable bidirectional far-field emission of a dipole emitter placed close to the nanoblock. Thanks to the wealth of multipolar modes in Si nanoblocks, the calculated directionality can reach over 25 dB. We reveal that a variety of mechanisms can exist within a single nanoblock, which contribute to the high directionality of the outcoming light. Briefly, featuring electric and magnetic dipolar resonances, zero-backward scattering of a Si nanoblock naturally appears when Kerker condition is satisfied. One the other hand, when coupled with a localized emitter, unidirectional backward emission can be achieved if the second Kerker condition for near-field excitation is satisfied. In addition, Si nanoblick can also behave as a detuned electric dipole, interfering destructively with the source dipole on the backwards, thus rendering unidirectional forward emission from the coupled system. Due to the different working principles of unidirectional forward and backward emission, their directionalities exhibit distinct dependencies on the local positions of the point emitter. Such property offers more possibilities for directional emission manipulation. Our study can be beneficial to the applications targeting the innovative nanophotonic devices, integrated optical nanocircuits and quantum communication. 

## 2. Materials and Methods

The three-dimensional, finite-difference, time-domain method (FDTD) was employed for the optical simulations. A total-field scattered field (TFSF) source was used to realize the planewave incidence and analyze the scattering properties of the Si nanoblocks. For near-field excitations, a broadband point electric dipole source representing a single emitter was located in the vicinity of the nanoblock to excite the multipolar resonances. The far-field radiation in the free space was obtained by recording the fields component in a closed box monitor, and near-to-far-field transformation (NTFF) was performed to these fields [30]. In all the FDTD calculations, a perfectly matched layer boundary was used, and the mesh size in the vicinity of the antenna was set to 2 nm for all cases. The refractive indices of silicon were taken from Palik [31] and the whole system is in the free space with air as the surrounding medium.

The Cartesian electric and magnetic dipole, and quadrupole moments of the nanoblocks under plane wave excitation were calculated using the standard expansion formulas [32]:

electric dipole moment:

(1) $$P_{\alpha }=\frac{1}{i\omega }\int J_{\alpha }(r)d^{3}r$$

magnetic dipole moment:

(2) $$M_{\alpha }=\frac{1}{2c}\int {\left[r\times J(r)\right]}_{\alpha }d^{3}r$$

electric quadrupole moment:

(3) $$Q_{\alpha ,\beta }^{E}=\frac{1}{i2\omega }\int [r_{\alpha }J_{\beta }(r)+r_{\beta }J_{\alpha }(r)-\frac{2}{3}\delta_{\alpha ,\beta }(r\cdot J(r))]d^{3}r$$

magnetic quadrupole moment:

(4) $$Q_{\alpha ,\beta }^{M}=\frac{1}{3c}\int [(r\times J{\left(r\right)}_{\alpha }r_{\beta }+(r\times J{\left(r\right)}_{\beta }r_{\alpha })]d^{3}r$$

where *ω* is the angular frequency, *c* is the speed of light, J is the induced current, r the position vector with the origin at the center of the nanoblock, and *α*, *β* = x, y, z.

The scattered electric far field resulting from the combination of these induced multipoles can be calculated by [13,27]

(5) $$E(r)=\frac{k_{}^{2}}{4\pi \varepsilon_{0}}\frac{e^{ikr}}{r}\{\hat{n}\times (P\times \hat{n})+\frac{\left(M\times \hat{n}\right)}{c}+\frac{ik}{6}\hat{n}\times (\hat{n}\times Q^{E}\hat{n})+\ldots \}$$

in which ***P*** (***M***) is the electric (magnetic) dipole moment, *Q^E^* is the electric quadrupole tensor.

A direct transfer of the multipole decomposition results from far-field excitations to near-field excitations is usually inappropriate. However, the results obtained in the case of plane-wave excitations show a predictive character for excitations by a localized dipole emitter [13,33]. Therefore, multipole decomposition was also performed to obtain the induced multipolar modes of the Si nanoblock excited by a point dipole emitter, where the origin of the coordinate system was chosen to lie in the center of the nanoblock. When considering the far field emission of the coupled emitter-nanoblock system, Formula (5) has to be revised accordingly to include the emission from the source emitter. The key difference for the near-field excitation configurations is the crucial role of the coherent interference between the induced multipoles in the nanoblock and the exciting dipole source, thus determining the total far-field emission characteristics (will be discussed later in detail). 

## 3. Results

**Figure 1:**
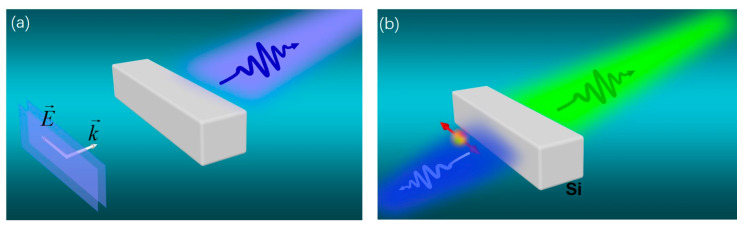
Schematic illusions of (**a**) unidirectional scattering of Si nanoblock under plane wave excitations as well as (**b**) switchable directive emission excited by a localized emitter. (**a**) The Si nanoblock was excited by a plane wave with polarization along the long axis of the nanoblock. When the Kerker condition is satisfied, a destructively interference in the backward direction occurs, leading to unidirectional forward scattering. (**b**) Sketch of a dipole emitter (denoted as the red arrow) in the vicinity of a silicon nanoblock in free space, showing forward and backward emission at different wavelengths.

The principle is schematically illustrated in Figure 1. This shows that a single Si nanoblock can act as a feasible and versatile ultracompact nanoantenna for effective direction control. Under plane wave illumination, unidirectional forward scattering can be realized at certain incident wavelength. Near-field resonant Si nanoblocks can modify the emission directivity of a localized driven source, leading to unidirectional emission into opposite directions depending on the emission wavelength.

**Figure 2:**
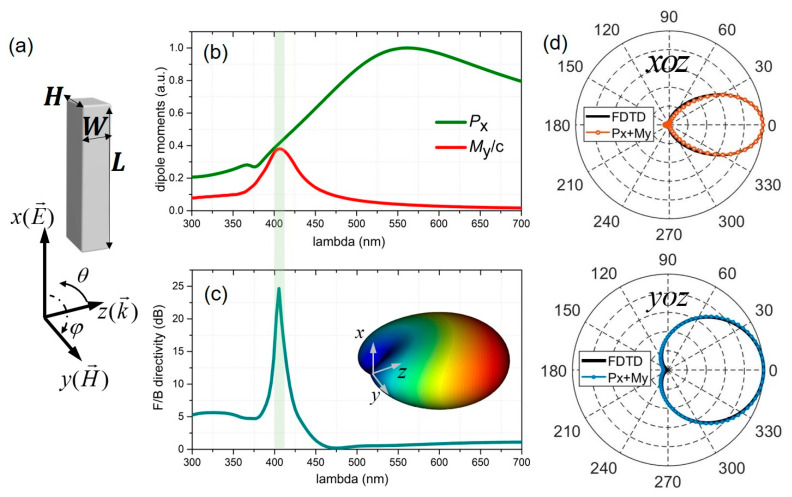
Unidirectional scattering of a single Si nanorod excited by a plane wave. (**a**) Sketch of the illumination configuration: a Si nanoblock with length (L), width (W) and height (H) illuminated by a plane wave injected from one side of the nanoblock (*E, H* and *k* indicate the electric field, magnetic field and propagation direction, respectively). (**b**) Induced electric dipole moment (*P*_x_) and the magnetic dipole moment (*M*_y_/c). (**c**) Forward-to-backward directivity as a function of wavelength. The inset shows the three-dimensional (3D) far-field radiation pattern. (**d**) The 2D radiation patterns on the xoz (top) and yoz (bottom) planes obtained by the combination of the electric dipole (ED) and magnetic dipole (MD) multipoles nicely reproduce the results calculated by finite-difference time-domain (FDTD). The geometric parameters used are L = 300 nm, W = 50 nm and H = 80 nm.

We first studied the scattering properties by imposing a plane wave on the Si nanoblock, with polarization parallel with the length of the nanoblock (Figure 2a). In such a scenario, the dominant multipoles contribute to the total scattering, as shown in the multipolar expansion results (Figure 2b), are the induced electric dipole (ED) and the magnetic dipole (MD). Figure 2c shows the far-field forward (FW) to backward (BW) directivity, defined as $F/B=10\log_{10}\left({\left|E_{far}(\theta =0^{\circ },\varphi =0^{\circ })\right|}^{2}/{\left|E_{far}(\theta =180^{\circ },\varphi =0^{\circ })\right|}^{2}\right)$, where $E_{far}$ is the amplitude of the radiated farfield, and φ,θ are the azimuthal and polar angle, respectively. It can be found that when dominant multipolar moments satisfy the Kerker condition *P_x_ = M_y_/c*, unidirectional emission can be realized along the +z direction. This can be confirmed by the observation of the highest *F/B* directivity at around 405 nm. Figure 2d depicts the 2D far-field scattering patterns on the xoz and yoz planes. These radiation patterns are reproduced using the analytical expression (Equation (5)) obtained by the combination of the dominant dipolar moments (***P*** and ***M***), and are superimposed with the FDTD calculations (solid lines), showing good agreement.

**Figure 3:**
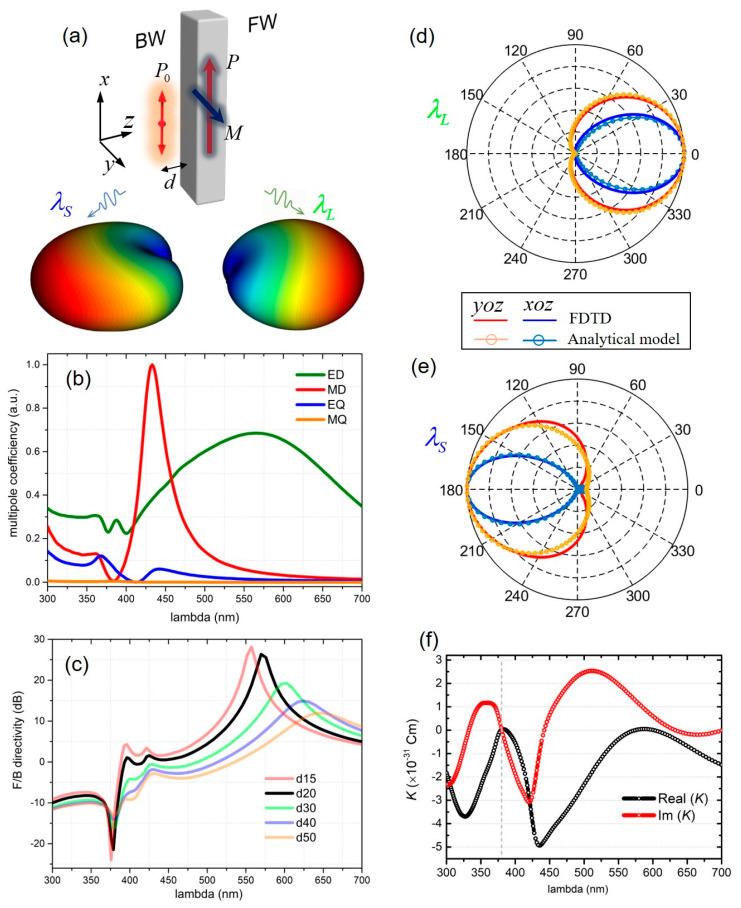
Concept of the switchable directive all-dielectric antenna. (**a**) The Si nanoblock is excited by a nearby electric dipole emitter with distance *d* of 20 nm away from the silicon surface. The point dipole excitation results in the induced multipoles (the induced electric and magnetic dipole moments are denoted as **P** and **M**) inside the nanoblock. 3D emission patterns calculated with FDTD show that at different wavelengths (*λ*_L_ and *λ*_S_), the light emits into the far-field forward (FW) and backward (BW) directions, respectively. (**b**) The multipole decomposition under the dipole excitation. Only the non-negligible multipole coefficients are shown. (**c**) F/B directivities as a function of wavelength for various emitter-nanoblock distances. 2D radiation patterns on the xoz plane and yoz plane calculated by the FDTD as well as the analytical model at emission wavelengths of (**d**) *λ*_L_ and (**e**) *λ*_S_. (**f**) Real and imaginary components of *K* are defined in Equation (7). Vertical dashed lines denote the wavelength *λ*_S_ that satisfies the generalized second Kerker condition for the near field excitation. The geometric parameters used are L = 300 nm, W = 50 nm and H = 80 nm. The two concerned wavelengths are *λ*_L_ ~ 564 nm and *λ*_S_ ~ 378 nm.

Next, we investigate the emitter-antenna coupled system and the corresponding far-field radiation characteristics. As shown in Figure 3a, a single excitation dipole source is oriented along x-direction and is located 20 nm away from the nanoblock surface at its symmetry axis. The decomposed induced electric dipole (ED), magnetic dipole (MD), electric quadrupole (EQ) and magnetic quadrupole (MQ) are shown in Figure 3b. Comparing the results of Figure 2b and Figure 3b clearly shows that near-field excitations, e.g., by a close-by dipole emitter, have an important impact on the excited multipolar resonances [17,34]. The non-negligible EQ contribution displays an especially resonant behavior at the operation short wavelength, indicating that the EQ mode plays an important role for the functionality of this directional emission.

The calculated F/B directivity shows distinct two extreme values at a short and long wavelength, respectively (Figure 3c, black curve for *d* = 20 nm). These two cases of unidirectional emission can be identified with unique mechanisms involving different multipolar modes, which will be discussed in detail in the following paragraphs.

At the longer wavelength of *λ_L_* around 560 nm, it is shown that the Si nanoblock supports the induced ED mode dominantly, from the multipolar expansion in Figure 3b. The induced ED of the Si nanoblock is coupled with the source electric dipole, composing a detuned-dipoles system, that strengthens the emission in the FW direction while totally suppressing the BW emission [17,35]. The dipole–dipole model provides an analytical expression for the time-averaged far-field Poynting vector as [36]

(6) S(r,θ,φ)=ω3k32π2ε0c2r2[|P0|2+|P|2+2Re(P0P∗eiklcosθ)](1−sin2θcos2φ)er

The power radiated in the ±z direction is proportional to cos^2^[(Δ*φ* ± *kl*)/2] if two oscillating dipoles have comparable amplitudes [28,37]. Herein, Δ*φ* = *φ*1 − *φ*2 is the phase difference between the two dipole moments; *k* is the wavelength vector; and *l* is the dipole–dipole separation distance. The electric dipole moment *P*_0_ of the source emitter is initially *P*_0_ = 1.49 × 10^−31^ Cm, and the induced dipole moment is calculated to be *P* = 1.5 $e^{0.844i\pi }\times 10^{-31}$ Cm. Due to the equal amplitudes of the dipole moments (|*P*_0_|/|*P*| = 0.993) and the phase delay as Δ*φ* − *kl* = −0.997 π, there is an almost complete suppression of the radiation in the −z direction, leading to the strongest FW emission directivity of up to 26 dB. The validity of the dipole-dipole coupling model is also confirmed with the FDTD calculations, which are shown as 2D plot of the far-field emission pattern on xoz and yoz planes in Figure 3d.

On the other hand, the emission light at short wavelength *λ_S_* around 380 nm undergoes a different pathway to the far field, because the driven source generates electric and magnetic dipolar moments as well as higher-order moments simultaneously. The resulting electric far-field comes from the interference of the contributing induced multipoles and the source dipole [9,10,20]: $E_{far}(r)=E_{P_{0}}(r)+E_{P}(r)+E_{M}(r)+E_{Q^{E}}(r)$. As such, Equation (5) should be revised to include the contribution of the source dipole. Taking the center of the nanoblock as the origin of the coordinate system, the electric far-field produced by the source dipole displaced at a distance *d* from the nanostructure on the Z-axis can be expressed as $E_{P_{0}}(r)=\frac{k_{}^{2}}{4\pi \varepsilon_{0}}\frac{e^{ikr}}{r}\hat{n}\times (P\times \hat{n})e^{-ikd\cos\theta }$ with a modified phase term exp(−i*kdcosθ*) [9]. By considering the far-field emission of the coupled system containing a source dipole moment $P_{0}=P_{0}\hat{x}$, an induced electric dipole moment along the X-axis $P_{x}$, an induced magnetic dipole moment along the y-axis $M_{y}$ and an induced electric quadrupole moment on the x−z plane $Q_{xz}$, the forward emission reads as

(7) $$S\propto \frac{k_{}^{4}}{4\pi \varepsilon^{2}{\left|E_{inc}\right|}^{2}}{\left|P_{0}e^{-ikd}+P_{x}+\frac{M_{y}}{c}-\frac{ik}{6}Q_{xz}\right|}^{2},$$

where $\left|E_{inc}\right|$ is the source dipole radiation intensity. We define $K=P_{0}e^{-ikd}+P_{x}+\frac{M_{y}}{c}-\frac{ik}{6}Q_{xz}$, and plot the wavelength dependence of the real and the imaginary components of *K* in Figure 3f. It should be noted that *K* = 0 in fact meets the generalized second Kerker condition for the near field exciation [9,33]. Our results hence come to the same conclusion as the previous reports [9,10], demostrating that while the conventional Kerker conditions are well-established when exciting the nanostructures with a plane wave, the concept can be extended to the near field by exciting the all-dielectric nanostructure with an electric dipolar emitter. As a result, the minimized FW emission is achieved, which is exactly what we observed at *λ_S_*, corresponding to the largest negtive F/B directivity in Figure 3c. FDTD method and the analytical model are also compared and they show a good agreement in the 2D radiation patterns in Figure 3e.

To this end, we have clarified that the excitation of multipolar modes within the single Si nanoblock by a source dipole empowers one to switch the directivity for different emission wavelengths. The two opposite directivities come from the distinct optical interferences between source dipole and the coherently excited multipoles. Therefore, the resulting FW and BW directivities have different dependencies on the emitter-nanoblock distance, since the distance *d* has impacts on both the phases and the magnitudes of the induced multipoles. Figure 3c presents the F/B directivity for various distance, *d*. It clearly shows that as distances increase, the directivity dramatically decreases due to the reduced near-field coupling strength between the source emitter and the Si nanoblock. It also shows that the spectral positions where the FW directivity occurs are largely affected by the emitter–nanoblock distances for the reason of such distance having a strong influence on the phase delay relationship in the dipole–dipole interaction system. By contrast, only a small wavelength drift is revealed where the largest BW directivity takes place. This is because the BW directivity originates from the interplay of the source emitter and the induced multimodal resonances. In this situation, the excitations of these resonances cause the scattered fields of the nanoblock that cancel the fields generating from the source in the forward direction, which could be less affected by the emitter–nanoblock distance. 

**Figure 4:**
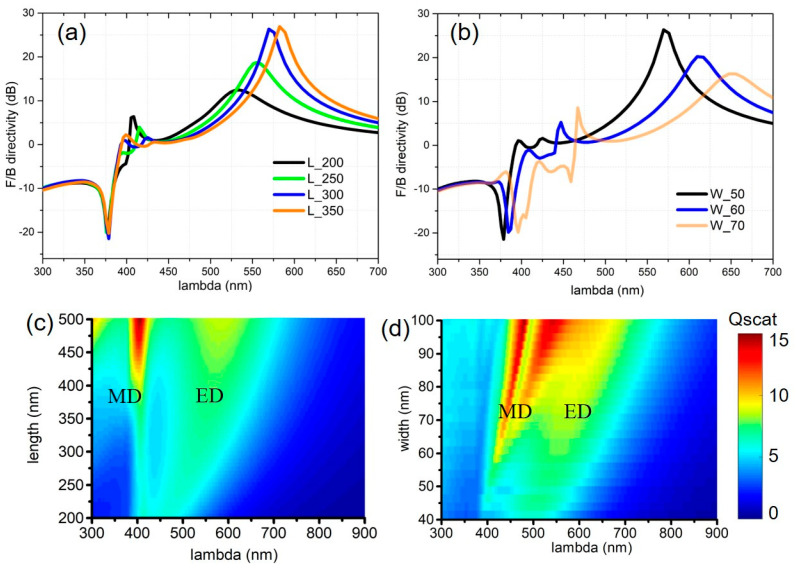
Structural dimension effects on the emission directivity. F/B directivities as a function of wavelength for (**a**) various lengths and (**b**) widths of the nanoblock. 2D map of evolution of the electromagnetic resonances with respect to the (**c**) lengths and (**d**) widths of the nanoblock as a function of the wavelength. The scattering efficiency is defined as the ratio of the scattering cross section to the physical cross section.

In the following, we will investigate the nanoblock’s structural dimension effects on the emission directivity. Figure 4a shows that the length of the nanoblock can drastically change FW directivity, while it has nearly no impact on BW directivity. This can be understood by investigating the spectral features of the induced multipole modes determined by structural dimensions. A two-dimensional map of the calculated scattering efficiency is shown in Figure 4c, clearly manifesting the broadband ED mode red-shifting with the increasing length and the narrowband MD mode shifting very little with changes in the length. Considering that the magnetic resonances originate from the circular displacement current [1], resonant magnetic response at *λ* ~ 400 nm would become dominant when the effective half-wavelength inside the nanoblock (*λ*/2n_si_) is comparable to its width W (i.e., 50 nm), where n_si_ is the refractive index of Si estimated to be 4 in the visible range. Therefore, the MD resonance wavelength is almost independent of the length of the nanoblock. As a result, the BW directivity that results from the interreferences of source dipole and induced ED, MD and EQ remain almost unchanged with variation in the length of the nanoblock. On the contrary, by increasing the width of the nanoblock, both ED and MD resonances will redshift (Figure 4d), yielding FW and BW directivities change accordingly (Figure 4c). Our calculations also indicate that the height of nanoblock largely affects ED mode and has a relatively small effect on MD modes, similar to the length effects on the directivities.

**Figure 5:**
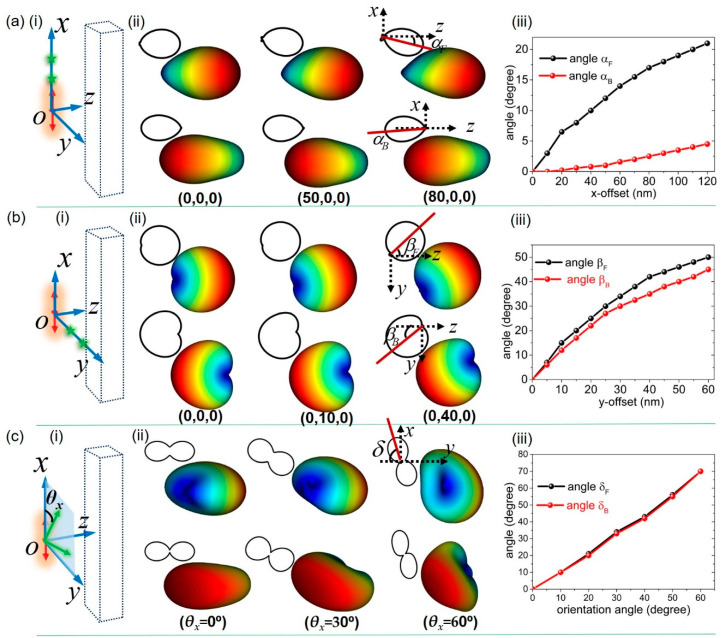
Far-field emission patterns dependencies on the configurations of the localized emitter and the nanoblock. (**a**) The source emitter has lateral displacement along x-axis. (i) sketch of the emitter-nanoblock configuration. The dipole emitter is denoted as a red arrow with initial position at the origin of the coordinates. Green star indicates the emitter at other offsetting positions. (ii) Side-views of far-field radiation patterns on the xoz plane and the top-left shows the corresponding cross-sections of the radiation patterns. The main lobe of the far-field emission shifts with increasing offset, with the maximal azimuthal angle denoted as α_F_ and α_B,_ respectively, for FW and BW emission. The bottom shows the coordinate positions of the source emitter. (iii) Dependence of the main lobe azimuthal angles on the offset values. (**b**) The source emitter has lateral displacement along y-axis (i). (ii) Side-views of far-field radiation patterns on the yoz plane and the corresponding cross-sections on the top-left. (iii) Dependence of the rotation angles on the offset values. (**c**) The source emitter has different orientation angles *θ*_x_, which are defined to be the angle between the dipole axis and x-axis (i). Perspectives of far-field radiation patterns and the cross-sections on the xoy plane on the top-left. (iii) Dependence of the rotation angles on the orientation angles.

We now examine the relative positions of the emitter respective to the nanoblock, because this could significantly influence the near-field coupling behaviors and consequently alter the emission directivities. Particularly, we show that the far-field radiations can be sensitive to lateral displacement of the emitter position in both x and y directions, as displayed in Figure 5. In all cases, the dipole emitter is placed 20 nm away from the nanoblock surface, and the optimal F/B directivities always occurred at *λ*_L_ and *λ*_S_, respectively, i.e., the same values as those in Figure 3. The coordinates are reset with its origin (0,0,0) as the initial position of the source emitter. Figure 5a(ii) shows that, as the lateral distance was increased along the x-direction, the forward emission features remained unchanged, but the maximal azimuthal angle (α_F_) of the angular patterns increased accordingly. When the displacement was increased to 80 nm, the main lobe of the forward emission was observed to be shifted by as much as 17°. This is understandable by considering the system as a pair of coupled dipoles with misplacement in the x-direction, resulting in the tilting far-field radiations depending on the offset values. In striking contrast to the FW directivity, the BW directivity of the emission is robust against the lateral offset along the x-direction. The insight is based on the fact that the wavelength *λ*_S_, corresponding to BW emission associates with the coherent near-field excitations of multipolar modes, and such displacements along the x-axis cause very small variations in the induced multipoles inside the nanoblock. Meanwhile, the light re-scattered by the nanoblock plays an essential role in the emission of such coupled system. As a result, it is seen in Figure 5a(iii) that a displacement of the dipole up to 120 nm changes the BW radiation pattern of the emission only slightly. 

Figure 5b shows the response of the emission to the displacement of the emitter along the y axis. This leads to the rotation of the radiation patterns on *yoz* plane while preserving the directivity. Shifting the dipole source to the positive y direction leads to a rotation of the pattern to the negative direction and vice versa. The beam-steering effect can be interpreted for the reason of asymmetric location of the source, and consequently the induced field in electric and magnetic multipoles also have an asymmetrical form [38]. The rotation angle *β* is dependent on the displacement value, and such behavior is shared by both FW and BW directional emission (Figure 5b(ii),(iii)). In a similar analogue, the orientation of the dipole source with the deviation angle with x-axis (denoted as *θ*_x_) can cause the rotation of far-field radiation on the *xoy* plane (Figure 5c). We emphasize that the effect of directivity preserves for all values of source offset up to the edge of the nanoblock and all value of deviation angles close to 90°. The combination of high-performance in unidirectional emission with the beam steering capability makes our results very promising for several applications in nanophotonics.

## 4. Discussion

In summary, a single dielectric silicon nanoblock was used to efficiently direct light from an incident plane wave or from localized light sources into a preferential direction. The interplays of the induced electric and magnetic multipoles inside the Si nanoblock were demonstrated to offer more degrees of freedom for controlling the directionality. Apart from the conventional forward unidirectional scattering as Huygens source upon plane wave illumination, our proposed subwavelength Si nanoantenna fed by a localized source emitter shows unique switchable directivity between forwards and backwards emission. Such switchable directionality involves optical interference effects when the light emitted from a localized emitter can follow different pathways to the far field, thanks to the coherently excited multipolar modes. The high-performance radiation directivity reaching over 25 dB can be operated in the visible spectral range and widely tuned via adjusting the structural dimensions of the nanoblock. We have also demonstrated the efficient beam-steering effect when the position and orientation of the localized source are well-controlled. The different working principles of forwards and backwards emission displaying different dependencies on the controlling parameters offers flexibility for directivity manipulation. The studies can also be combined with an optimization algorithm to optimize the design and increase the unidirectional spectral bandwidth [39,40]. Our findings suggest potential applications such as optical nanocircuitry and efficient integrated light sources.

## References

[1] Kuznetsov A.I., Miroshnichenko A.E., Brongersma M.L., Kivshar Y.S., Luk’yanchuk B. (2016). Optically resonant dielectric nanostructures. Science.

[2] Decker M., Staude I. (2016). Resonant dielectric nanostructures: A low-loss platform for functional nanophotonics. J. Opt..

[3] Kruk S., Kivshar Y. (2017). Functional Meta-Optics and Nanophotonics Governed by Mie Resonances. ACS Photonics.

[4] Yang Z.-J., Jiang R., Zhuo X., Xie Y.-M., Wang J., Lin H.-Q. (2017). Dielectric nanoresonators for light manipulation. Phys. Rep..

[5] Staude I., Pertsch T., Kivshar Y.S. (2019). All-Dielectric Resonant Meta-Optics Lightens up. ACS Photonics.

[6] Krasnok A.E., Maksymov I.S., Denisyuk A.I., Belov P.A., Miroshnichenko A.E., Simovski C.R., Kivshar Y.S. (2013). Optical nanoantennas. Physics-Uspekhi.

[7] Person S., Jain M., Lapin Z., Saenz J.J., Wicks G., Novotny L. (2013). Demonstration of zero optical backscattering from single nanoparticles. Nano Lett..

[8] Fu Y.H., Kuznetsov A.I., Miroshnichenko A.E., Yu Y.F., Luk’yanchuk B. (2013). Directional visible light scattering by silicon nanoparticles. Nat. Commun..

[9] Rolly B., Stout B., Bonod N. (2012). Boosting the directivity of optical antennas with magnetic and electric dipolar resonant particles. Opt. Express.

[10] Bidault S., Mivelle M., Bonod N. (2019). Dielectric nanoantennas to manipulate solid-state light emission. J. Appl. Phys..

[11] Staude I., Miroshnichenko A.E., Decker M., Fofang N.T., Liu S., Gonzales E., Dominguez J., Luk T.S., Neshev D.N., Brener I. (2013). Tailoring Directional Scattering through Magnetic and Electric Resonances in Subwavelength Silicon Nanodisks. ACS Nano.

[12] Rusak E., Staude I., Decker M., Sautter J., Miroshnichenko A.E., Powell D.A., Neshev D.N., Kivshar Y.S. (2014). Hybrid nanoantennas for directional emission enhancement. Appl. Phys. Lett..

[13] Zhang X.M., Zhang Q., Zeng S.J., Liu Z.Z., Xiao J.-J. (2018). Dual-band unidirectional forward scattering with all-dielectric hollow nanodisk in the visible. Opt. Lett..

[14] Feng T.H., Xu Y., Liang Z.X., Zhang W. (2016). All-dielectric hollow nanodisk for tailoring magnetic dipole emission. Opt. Lett..

[15] Guo R., Rusak E., Staude I., Dominguez J., Decker M., Rockstuhl C., Brener I., Neshev D.N., Kivshar Y.S. (2016). Multipolar Coupling in Hybrid Metal–Dielectric Metasurfaces. ACS Photonics.

[16] Arslanagic S., Ziolkowski R.W. (2018). Highly Subwavelength, Superdirective Cylindrical Nanoantenna. Phys. Rev. Lett..

[17] Cihan A.F., Curto A.G., Raza S., Kik P.G., Brongersma M.L. (2018). Silicon Mie resonators for highly directional light emission from monolayer MoS2. Nat. Photonics.

[18] Feng T.H., Zhang W., Liang Z.X., Xu Y. (2018). Unidirectional emission in an all-dielectric nanoantenna. J. Phys. Condens. Matter.

[19] Wiecha P.R., Cuche A., Arbouet A., Girard C., Colas des Francs G., Lecestre A., Larrieu G., Fournel F., Larrey V., Baron T. (2017). Strongly Directional Scattering from Dielectric Nanowires. ACS Photonics.

[20] Yang Y., Li Q., Qiu M. (2015). Controlling the angular radiation of single emitters using dielectric patch nanoantennas. Appl. Phys. Lett..

[21] Li J., Verellen N., Vercruysse D., Bearda T., Lagae L., Van Dorpe P. (2016). All-Dielectric Antenna Wavelength Router with Bidirectional Scattering of Visible Light. Nano Lett..

[22] Tian J., Li Q., Yang Y., Qiu M. (2016). Tailoring unidirectional angular radiation through multipolar interference in a single-element subwavelength all-dielectric stair-like nanoantenna. Nanoscale.

[23] Krasnok A.E., Miroshnichenko A.E., Belov P.A., Kivshar Y.S. (2012). All-dielectric optical nanoantennas. Opt. Express.

[24] Liu Y.G., Choy W.C.H., Sha W.E.I., Cho Chew W. (2012). Unidirectional and wavelength-selective photonic sphere-array nanoantennas. Opt. Lett..

[25] Lindfors K., Dregely D., Lippitz M., Engheta N., Totzeck M., Giessen H. (2016). Imaging and Steering Unidirectional Emission from Nanoantenna Array Metasurfaces. ACS Photonics.

[26] Shi T., Wang Y., Deng Z.-L., Ye X., Dai Z., Cao Y., Guan B.-O., Xiao S., Li X. (2019). All-Dielectric Kissing-Dimer Metagratings for Asymmetric High Diffraction. Adv. Opt. Mater..

[27] Xu C., Cheng K., Li Q., Shang X., Wu C., Wei Z., Zhang X., Li H. (2019). The dual-frequency zero-backward scattering realized in a hybrid metallo-dielectric nanoantenna. AIP Adv..

[28] Zhang X., Xiao J.-J., Zhang Q., Qin F., Cai X., Ye F. (2017). Dual-Band Unidirectional Emission in a Multilayered Metal–Dielectric Nanoantenna. ACS Omega.

[29] Ee H.-S., Kang J.-H., Brongersma M.L., Seo M.-K. (2015). Shape-Dependent Light Scattering Properties of Subwavelength Silicon Nanoblocks. Nano Lett..

[30] Zhang T., Xu J., Deng Z.-L., Hu D., Qin F., Li X. (2019). Unidirectional Enhanced Dipolar Emission with an Individual Dielectric Nanoantenna. Nanomaterials.

[31] Palik E.D. (1985). Handbook of Optical Constants of Solids.

[32] He Y., Guo G., Feng T., Xu Y., Miroshnichenko A.E. (2018). Toroidal dipole bound states in the continuum. Phys. Rev. B.

[33] Alaee R., Filter R., Lehr D., Lederer F., Rockstuhl C. (2015). A generalized Kerker condition for highly directive nanoantennas. Opt. Lett..

[34] Yang G., Niu Y., Wei H., Bai B., Sun H.-B. (2019). Greatly amplified spontaneous emission of colloidal quantum dots mediated by a dielectric-plasmonic hybrid nanoantenna. Nanophotonics.

[35] Ho J., Fu Y.H., Dong Z., Paniagua-Dominguez R., Koay E.H.H., Yu Y.F., Valuckas V., Kuznetsov A.I., Yang J.K.W. (2018). Highly Directive Hybrid Metal–Dielectric Yagi-Uda Nanoantennas. ACS Nano.

[36] Bonod N., Devilez A., Rolly B., Bidault S., Stout B. (2010). Ultracompact and unidirectional metallic antennas. Phys. Rev. B.

[37] Wen T., Zhang W., Liu S., Hu A., Zhao J., Ye Y., Chen Y., Qiu C.-W., Gong Q., Lu G. (2020). Steering valley-polarized emission of monolayer MoS2 sandwiched in plasmonic antennas. Sci. Adv..

[38] Krasnok A.E., Simovski C.R., Belov P.A., Kivshar Y.S. (2014). Superdirective dielectric nanoantennas. Nanoscale.

[39] Qin F., Zhang D., Liu Z., Zhang Q., Xiao J. (2019). Designing metal-dielectric nanoantenna for unidirectional scattering via Bayesian optimization. Opt. Express.

[40] Liu M., Xie Y., Feng T., Xu Y. (2020). Resonant broadband unidirectional light scattering based on genetic algorithm. Opt. Lett..

